# Metabolomic Study on Idiosyncratic Liver Injury Induced by Different Extracts of *Polygonum multiflorum* in Rats Integrated with Pattern Recognition and Enriched Pathways Analysis

**DOI:** 10.3389/fphar.2016.00483

**Published:** 2016-12-15

**Authors:** Chun-Yu Li, Can Tu, Dan Gao, Rui-Lin Wang, Hai-Zhu Zhang, Ming Niu, Rui-Yu Li, Cong-En Zhang, Rui-Sheng Li, Xiao-He Xiao, Mei-Hua Yang, Jia-Bo Wang

**Affiliations:** ^1^Institute of Medicinal Plant Development, Chinese Academy of Medical Sciences and Peking Union Medical CollegeBeijing, China; ^2^China Military Institute of Chinese Medicine, 302 Military HospitalBeijing, China; ^3^School of Pharmacy, Chengdu University of Traditional Chinese MedicineChengdu, China; ^4^Integrative Medical Center, 302 Military HospitalBeijing, China; ^5^Research Center for Clinical and Translational Medicine, 302 Hospital of People’s Liberation ArmyBeijing, China

**Keywords:** metabolomics, *Polygonum multiflorum*, idiosyncratic liver injury, stilbenes, pathway analysis

## Abstract

Currently, numerous liver injury cases related to a famous Chinese herb- *Polygonum Multiflorum* (Heshouwu in Chinese) have attracted great attention in many countries. Our previous work showed that Heshouwu-induced hepatotoxicity belonged to idiosyncratic drug-induced liver injury (IDILI). Unfortunately, the components and mechanisms attributed to IDILI of Heshouwu are difficult to determine and thus remain unknown. Attempts to explore puzzles, we prepared the chloroform (CH)-, ethyl acetate (EA)-, and residue (RE) extracts of Heshouwu to investigate IDILI constituents and underlying mechanisms, using biochemistry, histopathology, and metabolomics examinations. The results showed that co-treatment with non-toxic dose of lipopolysaccharide (LPS) and EA extract could result in evident liver injury, indicated by the significant elevation of plasma alanine aminotransferase and aspartate aminotransferase activities, as well as obvious liver histologic damage; whereas other two separated fractions, CH and RE extracts, failed to induce observable liver injury. Furthermore, 21 potential metabolomic biomarkers that differentially expressed in LPS/EA group compared with other groups without liver injury were identified by untargeted metabolomics, mainly involved two pathways: tricarboxylic acid cycle and sphingolipid metabolism. This work illustrated EA extract had close association with the idiosyncratic hepatotoxicity of Heshouwu and provided a metabolomic insight into IDILI of different extracts from Heshouwu.

## Introduction

The rising popularity of herbal medicines has promoted its use worldwide with a deep belief that herbs are safe because they are “natural” (Cheung, 2011; Stickel and Shouval, 2015). However, recent reports about herbs related hepatotoxicity from the literature are increasing (Navarro et al., 2014; Vuppalanchi et al., 2015). The *Polygonum multiflorum* (Heshouwu in Chinese), a popular traditional Chinese medicine, is widely used in hair-blacking and liver-and kidney-nourishing, even as dietary supplements. Nevertheless, numerous cases of Heshouwu and its preparations induced significant liver injury have emerged in recent years (But et al., 1996; Park et al., 2001; Mazzanti et al., 2004; Panis et al., 2005; Cardenas et al., 2006; Laird et al., 2008; Jung et al., 2011; Dong et al., 2014; Ma et al., 2014). The risks of Heshouwu-containing drugs have attracted much attention in many countries, and the regulations of those products had been strengthened by the Medicines and Healthcare Products Regulatory Agency (MHRA) of the United Kingdom and the China Food and Drug Administration (CFDA) in 2006 and 2013, respectively.

Unfortunately, the hepatotoxic chemicals and mechanisms attributed to Heshouwu induced liver injury remain unknown. It has been speculated that the toxicity may be related to stilbenes (Wu et al., 2012) or anthraquinones (Westendorf, 1993; Yu et al., 2011; Ma et al., 2015), which are main constituents of Heshouwu, but this hypothesis is in dispute and has not been proven. Although predictable liver toxicities are often discovered in preclinical and clinical testing for drugs, such testing does not apply to herbs because of their complex features (Willett et al., 2004). Furthermore, hepatotoxicity associated with Heshouwu is also extremely difficult to recognize or assess as it is idiosyncratic. A critical need exists to predict idiosyncratic drug-induced liver injury (IDILI) of Heshouwu and understand the mechanisms behind it. Currently, diagnostic methods of drug-induced liver injury (DILI), measuring levels of aspartate aminotransferase (AST) and alanine aminotransferase (ALT) etc. in blood samples, have been considered as the current “gold standard” for initial diagnosis and surveillance (Wang X. et al., 2012). However, these markers only offer a destination indicator and may lack sufficient sensitivity. Metabolomics, a powerful technology, could systematically assess metabolic profiles in easily accessible biofluids and biomarker discovery. Because this is a noninvasive technique, many diagnostic biomarkers and pathways of the organism can be tracked prior to toxicant exposure or during disease. Metabolomics has been successfully employed to identify specific biomarkers of toxicant exposure, which eventually could be used for early warnings of liver injury in clinic (Weiss and Kim, 2011; Xue et al., 2014; Rofiee et al., 2015).

In the light of the characteristic advantages of metabolomics, we hypothesized that metabolomics could detect the idiosyncratic hepatotoxicity biomarkers after exposure to constituents in Heshouwu. Therefore, in this study, UHPLC-MS-based metabolic approach was performed to investigate toxic components related to IDILI of Heshouwu. Moreover, to dig the underlying mechanism, specific biomarkers and pathways were discovered by pattern recognition and enriched pathways analysis.

## Materials and Methods

### Chemicals, Reagents, and Materials

Acetonitrile (HPLC grade) was purchased from Merck (Darmstadt, Germany); Formic acid and methanol were HPLC grade, and obtained from Fisher Chemicals (Pittsburg, PA, USA); water was purified by a Millipore’s ultrapure water system (Millipore, Bedford, MA, USA). Lipopolysaccharide (LPS) and sodium pentobarbital (Cat#P3761) were purchased from Sigma Chemical Company. LPS Derived from *Escherichia coli*, 055:B5 (Lot#113M4068V). Assays kits for the detection of serum alanine ALT and AST were purchased from Jiancheng Biological Technology, Co., Ltd (Nanjing, China). All other reagents and solvents were of the highest grade commercially available. The dry roots of *P. multiflorum* Thunb. (BN.13101701, Hubei, China) were purchased from Beijing Lüye Pharmaceutical Company and voucher specimen was deposited in China Military Institute of Chinese Medicine. *P. multiflorum* was verified to have met the standards specified by Chinese Pharmacopoeia.

### Sample Preparation for Different Extracts of *P. multiflorum*

The dried raw Heshouwu extracts were prepared through extraction eight times with a 50% ethanol-water solution by virtue of the cold soaking method, which was repeated two times for 48 h each time. The extracted liquid was mixed, filtered, and then concentrated under reduced pressure and dried under vacuum. Then the crude extract was diluted with deionized water to a suitable concentration. The diluted extract was then extracted with an equal volume of chloroform five times and subjected to vacuum drying to obtain the chloroform extract (CH). The ethyl acetate extract (EA) was obtained by further extracting the remaining aqueous fraction with an equal volume of ethyl acetate nine times. The remaining ethyl acetate-extracted aqueous portion was evaporated to obtain the Heshouwu residue (RE).

### Animals, Administration, and Sample Collection

Male Sprague-Dawley rats (180-250 g) were obtained from the Laboratory Animal Center of the Academy of Military Medical Sciences (License No. SYXK 2007- 004, Beijing, China). Animals received food and water *ad libitum* under standard husbandry conditions (25 ± 2°C temperature, 50-60% relative humidity and 12 h photoperiod) for 1 week of acclimatization. All procedures on animals and their care complied with the Guiding Principles for the Care and Use of Laboratory Animals of China and Institutional Animal Care and Use Committee of 302 hospital of PLA. The assessment of idiosyncratic hepatotoxicity was based on our previously reported rat model, which was modified from the literature. Briefly, the animals were intragastrically administered different extracts of Heshouwu or an equivalent volume of normal saline, followed by tail vein injection of LPS (2.8 mg/kg, Sigma) or normal saline 3 h later. At 7 h after the injection of LPS, rats were anesthetized with sodium pentobarbital (50 mg/kg, i.p.). Blood was collected from the inferior vena cava by the syringe containing sodium citrate (0.38% final concentration) after a midline abdominal incision, and the liver was removed. The isolated livers were utilized for histopathological examination and theplasma samples separated from the collected blood were applied for analysis of AST and ALT activities.

### Plasma ALT and AST Activities and Histopathological Assessment

After centrifugation (3,000 rpm, 10 min), plasma ALT and AST activities were determined according to the procedure of microplate assay kits. The left lateral liver lobes obtained were fixed in 10% neutral buffered formalin for at least 72 h before being processed for histologic analysis. Paraffin-embedded sections were cut at a 4 μm thickness and stained with hematoxylin and eosin for microscopic examination.

### Sample Preparation for Metabolomics

Two hundred microliter thawed plasma samples and 600 μL methanol were transferred to a 1.5 mL polypropylene tube andcentrifuged at 10,000 rpm for 10 min at 4°C. Supernatants were collected to filter through a syringe filter (0.22 μm).

### Chromatography and Mass Spectrometry Condition

Chromatographic analysis was performed in an Agilent 6550 iFunnel Q-TOF LC/MS (Agilent Technologies, USA). A ZORBOX RRHD C18 analytical column (100 mm × 2.1 mm., 1.7 μm, Agilent Technologies, USA), maintained at 30°C, was used for chromatographic separation. The sample sequence was random and 4 μL aliquot of each sample was injected into the column. The optimal mobile phase consisted of solvent A (0.1% formic acid in water) and solvent B (0.1% formic acid in acetonitrile). The flow rate was 0.3 mL/min with a linear gradient used was as follows: 0-1 min, 5% B; 1-9 min, 5 to 40% B; 9-19 min, 40 to 90% B; 19-21 min, 90 to 100% B; 21-25 min, 100% B. A 5-min post run time was inserted before the next sample. To ensure the stability and repeatability of the systems, a quality control (QC) sample was employed to optimize the condition of UHPLC-Q-TOF/MS, as it included majority of the whole plasma samples’ information. All the samples were holded at 4°C during the experiment.

The mass spectrometry was performed by the Agilent 6550 Q-TOF/MS with an electrospray ionization source (ESI) in both positive and negative mode. The optimal conditions of analysis were set as follows: dry gas flow rate was 11 L/min; dry gas temperature was set at 200°C in negative ionization mode and 225°C in positive ionization mode. Sheath gas flow rate 11 L/min and sheath gas temperature was 350°C. Electrospray capillary voltage was 3.0 kV in negative mode and 4.0 kV in positive mode. Nozzle voltage was 500 V in negative mode and the same as in positive mode. Nebulizer pressure was set to 35 psig (negative) and 45 psig (positive). MS data were gathered in the full scan mode from m/z 80-1200 with a scan rate of 1 spectra/s. For internal mass calibration during the MS analysis, reference masses 112.9856 (TFANH4, [C2H4O2NF3-NH4]-) and 1033.9881 (TFANH4+HP-0921, [C20H22O8N4P3F27-NH4]-) were used in negative mode, and 121.0509 (Purine, [C5H4N4+H]+) and 922.0098 (HP-0921, [C18H18O6N3P3F24+H]+) were used in positive mode.

### Data Processing and Pattern Recognition Analysis

All data were pre-processed with Profinder. For molecular feature extraction, up to 2000 compounds with peak height above 300 counts were extracted, the binning and alignment settings were “RT window” = 0.3 min and “mass window” = 35 ppm. Then the data was introduced into the SIMCA-P 13.0 version, which was used for multivariate statistical analyses including principal component analysis (PCA) and orthogonal partial least squares discriminant analysis (OPLS-DA) (Zhang et al., 2016).

The variable influence on the projection (VIP) parameter was applied to choose variables that have the most significant contribution in discriminating between metabolomic profiles of experimental animal groups in an OPLS-DA model. Only variables with VIP values >1 and |*p* (corr)|≥ 0.5 were selected and used for further data analysis.

### Biomarker Identification and Metabolic Pathway Analysis

Compounds were selected as potential biomarkers with significant changes among groups (*p*-value < 0.05 and folder change > 1.5). They were identified by Agilent MassHunter PCDL Manager software, with KEGG^1^ database. To further identify the metabolic pathways, the pathway analysis of potential biomarkers was executed with MetaboAnalyst 3.0^2^ on the basis of the te pathway library of Rattus norvegicus (rat). When the test p value is less than 0.05, statistical differences are considered significant.

## Results

### Chemical Compositions Determination of the Different Extracts of *P. multiflorum*

The chemical compositions of different extracts of Heshouwu were determined by UHPLC approach, which was showed in Supplementary Material. The results indicated that there were a roughly separation within the CH, EA, and RE extracts. The EA extract contained mostly stilbenes (TSG) and trace anthraquinone glycoside (emodin-8-*O*- glucoside). The CH extract contained only anthraquinones (mostly emodin-8-*O*- glucoside and emodin), and the RE extract contained neither anthraquinones nor stilbenes. Supplementary Figure S1 shows the representative UHPLC chromatograms of mixed standards (Supplementary Figure S1A) and the different Heshouwu extracts (Supplementary Figures S1B–D).

### Liver Functional and Histologic Changes of Different Extracts of *P. multiflorum*

**FIGURE 1:**
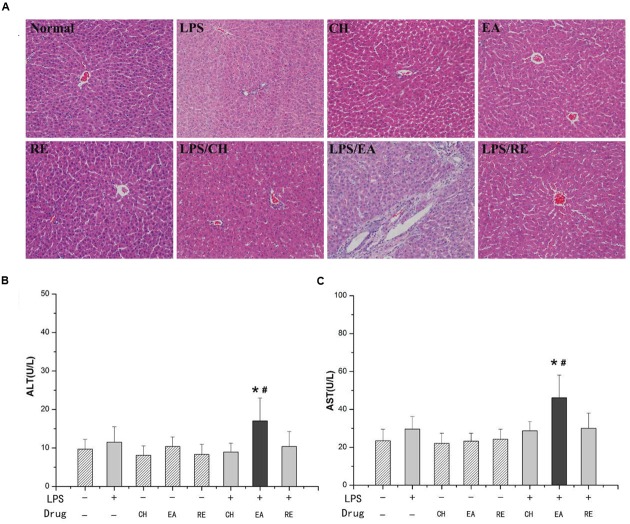
**(A)** Representative microphotographs of liver sections isolated from rats. HE staining (×200). **(B,C)** Influence of co-treatment with lipopolysaccharide (LPS) and different extracts of *Polygonum multiflorum* on plasma aminotransferase (ALT) and aspartate aminotransferase (AST) activities. For LPS-treated groups, “-” and “+” represent intravenous administration of saline and 2.8 mg⋅kg^-1^ of LPS, respectively, and for drug-treated groups, “-”,“chloroform, CH”, “ethyl acetate, EA”, and “residue, RE” represent intragastric administration of saline and “CH”, “EA”, and “RE”, respectively. Saline-treated group served as a control group and LPS-treated group served as a model group. *n* = 10, x ± s. ^∗^*P* < 0.05 vs. control group; **^#^***P* < 0.05 vs. model group.

Treatment of rats with isolated extract fractions (EA, CH, and RE) alone did not induce significant increases in plasma ALT and AST activities compared with normal group (*P* > 0.05). Likewise, no increase in ALT and AST activities were evident with the dose of LPS used in the study (*P* > 0.05). However, ALT and AST activities were remarkably increased in rats cotreated with LPS and EA extract. In contrast, administration of CH or RE extract to LPS-treated rats did not result in markedly increased plasma ALT and AST compared with both the normal and LPS control groups (**Figures 1B,C**).

Livers from saline-treated (control), EA-treated, CH-treated and RE-treated rats had no or minimal histopthological changes. Treatment with LPS (2.8 mg/kg, i.v.) alone caused slight infiltration of inflammatory cells in portal area but no evident hepatocytes injury. Co-treatment with LPS (2.8 mg/kg, i.v.) and EA caused hepatocyte focal necrosis, loss of central vein intima and a large number of inflammatory cell infiltration in portal areas that had high plasma ALT and AST activities (**Figure 1A**). These data suggested that the EA extract, but not the CH or RE extract, probably plays the major role in the pathogenesis of the idiosyncratic hepatotoxicity of Heshouwu.

### Multivariate Statistical Analysis of the Metabolomics Data

**FIGURE 2:**
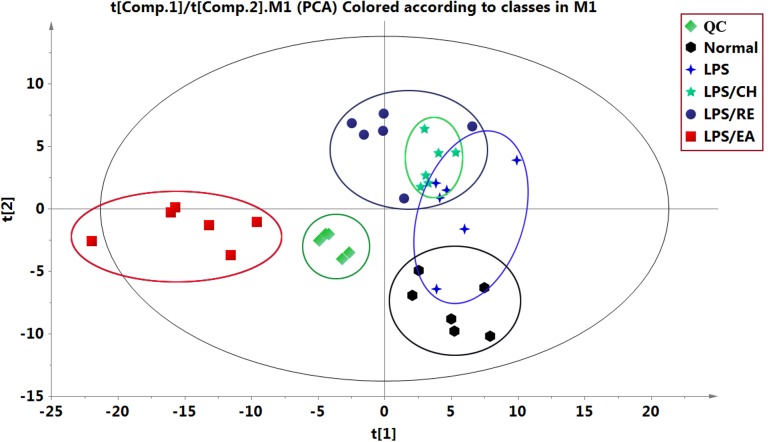
**Principal component analysis (PCA) score plots of different extracts of *P. multiflorum* by UHPLC-MS in negative electrospray ionization source (ESI) mode**.

Using UHPLC system, global metabolic profiles in both negative and positive ion modes were analyzed. For better visualizing the subtle differences among these complex data sets, multiple pattern recognition methods were applied to differentiate the phenotype from plasma metabonome of rats. Firstly, an unsupervised PCA statistical method was employed to study the metabolic differences. The score plots of PCA analysis derived from data of ESI- mode and ESI+ mode are shown in **Figure 2** and Supplementary Figure S2, respectively. Each point represents an individual sample in the PCA scores. The PCA results are displayed as score plots demonstrating the scatter of the samples, which suggest compositionally different metabolomes when dispersed andsimilar metabolomic compositions when clustered together. The different plasma samples could be divided into different blocks by the PCA scores plot, indicating that the metabolic profiles have changed. The QC samples clustered closely in both ESI- and ESI+ score plots, indicating the stability of the LC/MS system throughout the whole analysis. An obvious separation trend can be observed among the control, LPS, LPS/CH, LPS/EA, and LPS/RE groups in both PCA model, demenstrating there was a considerable metabolite difference among the five groups. Besides, it should be noted that there is a small overlapping among the control, LPS, LPS/CH, and LPS/RE groups, whereas LPS/EA group is far away from the remaining four groups, which indicate that changed metabolic pattern resulted from EA-induced liver injury may be significantly different from others.

**FIGURE 3:**
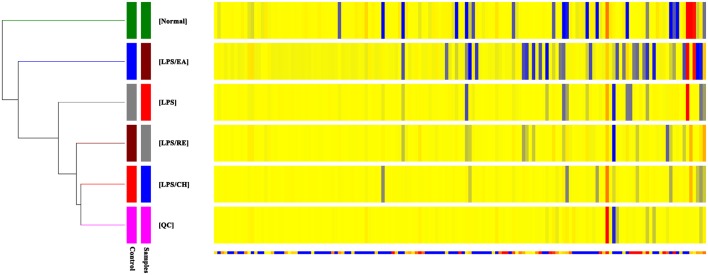
**Heatmap visualization for different extracts of *P. multiflorum***. Rows: samples; Columns: metabolites (ESI- mode).

To further investigate the metabolic changes among different groups, the clustering heatmaps were constructed based on the differential metabolites of importance, which were extracted with PCA analysis in negative ESI mode and in positive mode. As shown in **Figure 3** and Supplementary Figure S3, we found that that this model was capable of distinguishing LPS/EA group from control, LPS, LPS/CH, and LPS/RE groups.

**FIGURE 4:**
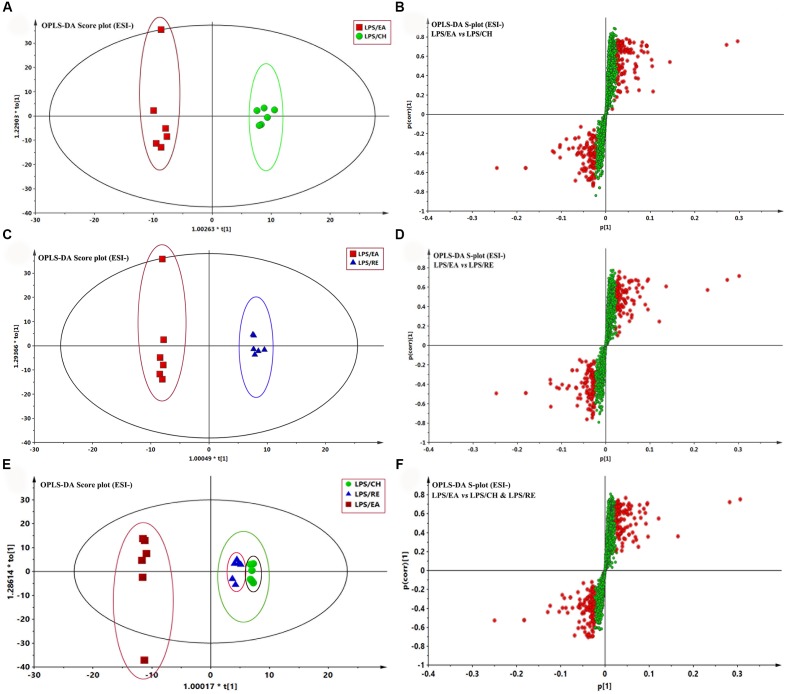
**Orthogonal partial least squares-discriminant analysis (OPLS-DA) analysis of the data generated from the ESI- mode**. S-score plots constructed from the supervised OPLS analysis of serum **(B,D,F)**, the axes that are plotted in the S-plot from the predictive component are *p*1 vs. *p*(corr)1, representing the magnitude and reliability, respectively. Metabolite ions with variable influence on the projection (VIP) value >1 were marked with a red square. **(A,B)** Displays the result of OPLS-DA model using the data from the LPS/EA and LPS/CH groups in ESI- mode. **(C,D)** Displays the result of OPLS-DA modelusing the data from the LPS/EA and LPS/RE groups in ESI- mode. **(E,F)** Displays the result of OPLS-DA model using the data from the LPS/EA, LPS/CH and LPS/RE groups in ESI- mode.

To highlight the metabolite differentials among LPS/CH, LPS/EA, and LPS/RE groups respectively, the supervised method, OPLS-DA, was used to classifiy or discriminate analyses, for its calculation yielded a significantly distinct degree of biological variation. The result of OPLS-DA model derived from data of ESI^-^ analysis is displayed in **Figures 4A-F**, respectively, suggesting that the metabolic profiles have changed. The analyst of OPLS-DA model using the data from the LPS/CH and LPS/EA groups are showed in **Figure 4A**. As **Figure 4A** reports, the score plot of OPLS-DA model was reliable. The LPS/EA group can be separated from LPS/CH group very clearly. The LPS/EA indicated good predictive ability with a R2X (cum) of 0.586, R2Y (cum) of 0.990, and Q2 (cum) of 0.661. Likewise, the OPLS-DA model was constructed based on the LPS/EA and LPS/RE data (**Figure 4C**). The LPS/EA group can be separated from LPS/RE group clearly. The R2X (cum), R2Y (cum), and Q2 (cum)were 0.631, 0.996, and 0.503, respectively. In addition, clear separation was observed among LPS/EA, LPS/CH and LPS/RE groups in the score plot. The LPS/EA group was clustered together and relatively far from other two groups, which were aggregated in the same region. It also indicated the trend of EA-induced liver injury in LPS-treated rats (**Figure 4E**). The values of R2X(cum), R2Y(cum), and Q2Y(cum) as 0.606, 0.812, and 0.502, respectively. The data analysis of ESI+ mode was also conducted and are shown in Supplementary Figure S4. All the parameters of PCA and OPLS-DA model are listed in Supplementary Table S1.

### Potential Biomarker Selection, Discovery, and Explanation

Potential markers were selected in accordance with their contribution to the correlation and variation within the data set, which were extracted from S-plots constructed following the OPLS analysis. In the S-plots, the variables farther away from the origin contribute significantly and are responsible for the separation between aforementioned groups and may therefore be viewed as potential biomarkers. In general, variables with a VIP value >1 were pre-selected as potential biomarkers. To decrease the risk of false positives in a choice of potential biomarkers, variables with |*p* (corr)|≥ 0.5 were chosen as the variables that were most related with the OPLS-DA discriminant scores. The variables that do not vary significantly are plotted in the middle and those that changed most significantly are plotted at the top or bottom of the S plot. The S-plots for LPS/EA vs LPS/CH, LPS/EA vs LPS/RE, LPS/EA vs LPS/CH& LPS/RE are shown in **Figures 4B,D,F**, respectively. The same process was performed for the data from the ESI**+** analysis (Supplementary Figure S4 and Table S1).

Then, compared with LPS/CH and LPS/RE groups, solo or together, the metabolites differed significantly (*P* < 0.05) in the LPS/EA group were selected as candidate biomarkers. The criteria were restricted to features with an average normalized intensity difference of 1.5-fold. Finally, the metabolites in the ESI+ and ESI- mode analyses were combined and subjected to further identification of their molecular formulas. All biomarkers were tentatively identified with the accurate mass charge ratio by the online METLIN database^3^.

Using the above mentioned approach, 412 metabolites were identified and were selected as the biomarkers. By applying this analysis process, the variables responsible for group separation were selected as potential biomarker candidates. After removal of the duplicate potential biomarkers, 21 variables were selected as the candidates of potential biomarkers in LC/MS (**Table 1**).

**Table 1:** Identification and trends of change for potential biomarkers.

No^b^	R.T. (min)	Biomarkers	Mass	Formula	Content variance^a^
ESI^+^					
1	0.94	L-Valine	117.0790	C5H11NO2	↓
2	1.09	Creatine	131.0695	C4H9N3O2	↓
3	1.09	Nicotinamide	122.0480	C6H6N2O	↑
4	3.97	epsilon-Caprolactam	113.0841	C6H11NO	↑
5	13.57	Sphingosine	299.2824	C18H37NO2	↓
6	14.86	Orthophosphate	97.9769	H3O4P	↓
7	15.85	UDP-*N*-acetylmuramoyl-L-alanyl-gamma-D-glutamyl-L-lysine	1007.2774	C34H55N7O24P2	↑
8	19.72	Sphinganine 1-phosphate	381.2644	C18H40NO5P	↓
9	19.89	Cortol	368.2563	C21H36O5	↓
ESI^-^					
1	0.84	Succinate	118.0266	C4H6O4	↑
2	0.85	D-Lombricine	270.0729	C6H15N4O6P	↓
3	0.93	Oxalosuccinate	190.0114	C6H6O7	↑
4	0.94	Urate	168.0283	C5H4N4O3	↑
5	0.94	gamma-Glutamyl-gamma-aminobutyraldehyde	216.1110	C9H16N2O4	↓
6	0.94	2-C-Methyl-D-erythritol 4-phosphate	216.0399	C5H13O7P	↓
7	0.98	*O*-Succinyl-L-homoserine	219.0743	C8H13NO6	↑
8	1.11	Caproic acid	116.0837	C6H12O2	↓
9	1.13	4-Pyridoxic acid	183.0532	C8H9NO4	↓
10	5.38	Phenol	94.0419	C6H6O	↑
11	9.69	Imidazole lactate	156.0535	C6H8N2O3	↓
12	19.83	beta-Nitropropanoate	119.0219	C3H5NO4	↑

*^a^Change trends of potential biomarkers in LPS/EA compared with LPS/CH and LPS/RE groups. ^b^Potential biomarkers identified both in positive and negative electrospray ionization source (ESI) mode. The levels of potential biomarkers were marked with (↓) down-regulated, (↑) up-regulated*.

## Discussion

Despite a significant increase of Heshouwu-induced hepaototoxity has been reported, they occur in a minority of patients in fact by means of the retrospective statistics of medicinal liver injury cases in 302 Hospital, so that they might be idiosyncratic liver injury based on integrated evidence chain-based identification of Chinese herbal medicine (Wang et al., 2015; Zhu et al., 2016). Whereas, the traditional toxicity evaluations showed that Heshouwu treatments caused hepatotoxicity requiring more than 50∼70 times of clinical equivalent dose with continuous administration for 4∼8 weeks (Wang T. et al., 2012; Li et al., 2013; Zhang et al., 2013), resulting in difficult evaluate the hepatotoxicity of herbal medicines. Moreover, predicting idiosyncratic hepatotoxicity reactions has been a formidable challenge. The LPS model has been employed to evaluate IDILI successfully for several drugs in humans, such as trovafloxacin, sulindac, halothane, chlorpromazine, monocrotaline, ranitidine, diclofenac, and amiodarone in toxicological experiment assessment on the basis of the inflammatory stress hypothesis (Waring et al., 2006; Shaw et al., 2010; Roth and Ganey, 2011; Poulsen et al., 2014). The previous research constructed Heshouwu idiosyncratic hepatotoxicity, based on LPS animal models, has manifested Heshouwu could induce acute liver injury in rats cotreated with LPS approximate to the clinical equivalent dose, and the animal model should make it possible to assess idiosyncratic hepatotoxicity of the herb (Li et al., 2015).

However, the idiosyncratic hepatotoxic material on the basis of Heshouwu is still unclear. Besides, the present studies also have controversial issue (Wang et al., 2014). Several studies have demonstrated that anthraquinones, e.g., emodin, are the major hepatotoxins in Heshouwu (Yu et al., 2011; Lin et al., 2015; Ma et al., 2015). Westendorf (1993) concluded that the herb toxicity might be associated with anthraquinones that form highly reactive anthrones in the colon, thus resulting in hepatotoxicity. Whereas, another report argued that the toxicity of Heshouwu does not depend on the content of anthranoid derivatives, on account of the stilbenes content was decreased and emodin content was increased after being processed, it may be correlated with the content of tetrahydroxystilbene glucosides (Wu et al., 2012). Results showed that when free anthraquinones (mainly emodin) are extracted with CHCl_3_, comparable to the dose of which in total extracts, didn’t induce liver damage in saline-treated rats and in LPS-treated rats. Furthermore, solo administration of either isolated EA extract or RE extract to normal rats failed to induce significant liver injury (**Figure 1**). Meanwhile, liver damage can be seen in a comparable dose of the EA extract and not in RE extract in LPS-treated rats, suggesting that EA extract was the main ingredient associated with Heshouwu induced idiosyncratic hepatotoxicity. The mechanism of IDILI is very complex, among which inflammation response plays a critical role. The inflammatory stress hypothesis has provided some of the first animal models of idiosyncratic hepatotoxicity (Roth and Ganey, 2011; Poulsen et al., 2014). Nontoxic dose of LPS precipitate modest inflammatory responses in mammals, resulting in increased susceptibility to toxicity from numerous hepatotoxic chemicals (Deng et al., 2009). These results demonstrated that modest inflammation response, triggered by a small dose of LPS, augments hepatotoxicity induced by EA extract not either CH or RE extract. Furthermore, UHPLC was used to confirm the chemical compositions of EA extract, 91% of which was stilbenes mainly containing 2,3,5,4’-tetrahydroxy transstilbene-2-*O*-β-glucoside (*trans*-SG) and its *cis*-isomer (*cis*-SG), the other was slight emodin-8-*O*-β-D-glucopyranoside. So EA extract might be idiosyncratic hepatotoxic constituents attributed to Heshouwu-induced IDILI. Furthermore, our recent finding indicated that *cis*-SG might be the major hepatotoxic component not either *trans*-SG or emodin-8-*O*-β-D-glucopyranoside in LPS-treated rats. This is the first report of idiosyncratic hepatotoxic substances in association with Heshouwu.

**FIGURE 5:**
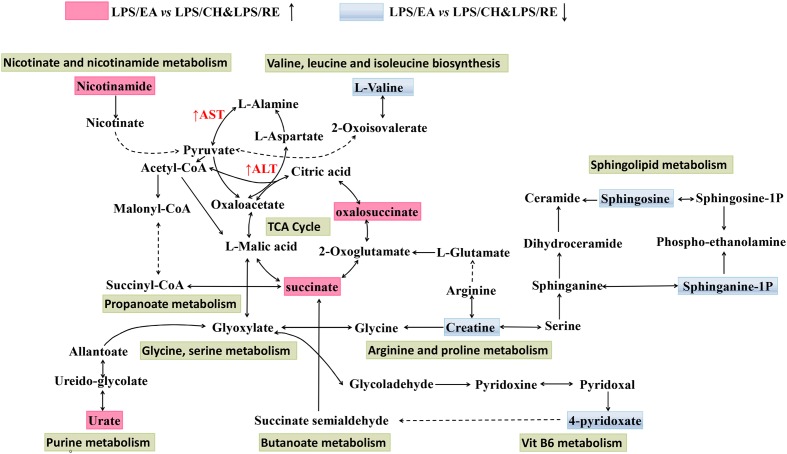
**Schematic diagram of the disturbed metabolic pathway related to LPS/EA treatment**. The notations are as follows: (↑) in red, metabolite higher in LPS/EA treated group than in LPS/CH and LPS/RE groups; (↓) in green, metabolite lower in LPS/EA treated group than in PS/CH and LPS/RE groups. The related metabolic pathways are cycled in a black box; ALT, glutamate pyruvate transaminase; AST, aspartate aminotransferase.

Metabolomics is generally a kind of phenotype-guided study, aiming to link phenotypic characterization of multifactorial diseases or drug toxicity with metabolomics profiles or changes (Ramautar et al., 2013; Frederich et al., 2016). In the present study, we discovered that EA extract was the main component associated with Heshouwu-induced idiosyncratic hepatotoxic phenotype, on the basis of the background of LPS-induced minimal inflammation. Without the LPS-induced background, neither EA nor the other parts of extracts displayed hepatotoxic phenotype. Comparisons between the LPS/EA vs. EA group and LPS vs EA group might be meaningful in explanation of the systematic perturbation of LPS or EA, but we did not focus on these issues. Thus, we emphasized the comparisons between EA/LPS group and the other extracts/LPS groups to find potential metabolomic biomarkers associated with idiosyncratic hepatotoxicity. Reports have shown that metabolomic approach based on the UHPLC-MS can be used as a robust tool to inspect the effects of hepatotoxic compounds (Weiss and Kim, 2011; Xue et al., 2014; Rofiee et al., 2015). Accordingly, UHPLC-MS based metabolomic technique was utilized used to investigate the alterations of plasma metabolite profiles in rats after combined exposure to LPS and with different extracts of Heshouwu. The data were analyzed by PCA showing an obvious separation among the samples. Especially, LPS/EA group was far away from the remaining four groups, suggesting EA-induced liver injury may be significantly different from others. The results revealed that this metabolomics can be utilized to characterize the metabolic perturbation of different extracts of Heshouwu to elucidate the toxic effects. Biomarkers, serving as early biochemical changes in drug induced hepatotoxicity, provided an opportunity to develop predictive biomarkers that could provide valuable insights about mechanisms of hepatotoxicity (Miao et al., 2016). Compared with LPS/CH and LPS/RE treated groups, 21 potential biomarkers related to liver injury, such as oxalosuccinate, succinate, creatine, and L-valine acid and so on, had been differentially expressed in LPS/EA group. The important plasma biomarkers related to liver injury were further analyzed and the possible metabolic pathway of EA-induced hepatotoxicity is displayed in **Figure 5**.

As shown in **Figure 5**, Supplementary Figure S5, and Supplementary Table S2 changes in tricarboxylic acid cycle (TCA cycle) and sphingolipid metabolism were prominent among the affected metabolisms in the LPS/EA group compared to LPS/CH and LPS/RE group.; nine out of 21 significant metabolites accountable for class discrimination were oxalosuccinate, succinate, Urate, 4-pyridoxic acid, sphinganine 1-phosphate, nicotinamide, sphingosine, L-valine, and creatine by schematic diagram of the disturbed metabolic pathway. In addition, the relative mean peak area of nine different metabolites was graphed in Supplementary Figure S6. The relative concentration of nine endogenous metabolites was significantly affected for LPS/EA treatment. By contrast, there was not significant change whether in normal group or in LPS group, LPS/CH group and LPS/RE group. They could be considered as potential markers for biological pathway analysis. Previous studies showed that one of the reasons for drug induced liver injury is the disturbance of the hepatocyte energy metabolism, particularly in the Krebs cycle (Mogilevskaya et al., 2006). In the experiment, TCA cycle may be most related to the hepatotoxicity of EA. The observed significant increase in the level of succinate and oxalosuccinate in LPS/EA treated rats revealed that dysfunction of energy metabolism and EA influenced the activity of mitochondrial enzymes related to the TCA cycle, leading to the disruption of energy metabolism in the liver (**Figure 5**). Succinate and oxalosuccinic are the intermediates in the TCA process, which are mainly dealt with in liver mitochondria. Furthermore, TCA cycle function also can be viewed as a bridge connected with other disturbed metabolic pathways. For example, succinate is an intermediate in TCA cycle, at the same time it is the metabolite of 4-Pyridoxate (Also in vitamin B6 metabolism pathway). Therefore, it was possible that EA affected the energy metabolism in liver mitochondria. Additionally, decreased plasma creatine levels were seen from LPS/EA treated rats. Metabolomic analysis also showed lower levels of creatine in subjects with steatosis (Kalhan et al., 2011). Creatine, found to be decreased in both the animal and human non-alcoholic fatty liver disease samples, has long been employed to evaluate possible liver dysfunction (Barr et al., 2010). The creatine-phosphocreatine system is essential for cellular energy transportation (Sun et al., 2012). Therefore, the decreased levels of serum creatine observed in the LPS/EA treated rats are consistent with disruption of the energy homeostasis.

In sphingolipids metabolism, sphingolipids play significant roles in membrane biology and provide many bioactive metabolites, such as ceramide, sphinganine, sphingosine, sphingosine 1-phosphate(S1P), that regulate cell function as intracellular and extracellular mediators (Gault et al., 2010; Nojima et al., 2015). As a sphingolipid metabolite, ceramide is metabolized to sphingosine and S1P by ceramidase and sphingosine kinases (Nixon, 2009). Sphinganine 1-phosphate (sphinganine 1-P) is structurally similar to S1P and is produced by the ATP-dependent phosphorylation of sphinganine by sphingosine kinase (Le Stunff et al., 2004). In this study, the decreased concentration of sphingosine and sphinganine 1-P in the LPS/EA group compared with LPS/CH and LPS/RE groups were observed. The concentration of sphingosine decreased in LPS/EA treated rats might be associated with ceramide accumulation that has also been demonstrated in models of hepatic I/R injury and liver transplantation *in vivo* (Bradham et al., 1997; Alessenko et al., 2002). In addition, plasma sphinganine 1-P levels were noted to be decreased after hepatic I/R injury and it has been reported to protect against both liver and kidney injury (Park et al., 2010).

Taken together, associated with the aforementioned inflammatory response activation involved in the pathogenesis of liver injury, interactions between the inflammatory response and metabolic systems might play a key role in the biological mechanism of EA extract induced idiosyncratic hepatotoxicity. However, some limitations still exist in this investigation. Despite the potential biomarkers of the idiosyncratic hepatotoxic mechanism of EA extract distinguished with other two extracts have been explored, but verification of these biomarkers and systematic clinical elucidation and evidence are still needed. Additionally, the material basis and exact biological mechanism responsible for the IDILI are also needed further exploration.

## Conclusion

Biochemistry and histopathology analyses indicated that EA extract might be the major hepatotoxic constituents of Heshouwu in LPS-treated rats, compared to CH and RE extracts. The comprehensive metabolomics approach based on UHPLC-MS was developed and successfully applied to explore the biological changes induced by different extracts of Heshouwu in LPS-treated rats. By virtue of the information extracted from the multivariate analysis, specific changes of metabolites among different extracts of Heshouwu were identified, and 21 key biomarkers which are responsible for the hepatotoxic compounds were discovered. Alterations in these metabolites primarily involved TCA cycle and sphingolipid metabolism pathways. This research not only provide systematic experimental basis for the major hepatotoxic constituents of Heshouwu firstly, but exemplifies that metabolomics could provide a promising way to elucidate hepatotoxic mechanisms of Heshouwu, thereby permitting a comprehensive understanding of systemic toxicity for traditional Chinese medicine.

## Ethics Statement

The study was approved by Experimental Animal Center of 302 Military Hospital, Beijing, China. The experiment complies with the animal welfare and ethics and it is approved. Male Sprague-Dawley rats (180-250 g) were obtained from the Laboratory Animal Center of the Academy of Military Medical Sciences (License No. SYXK 2007- 004). Vulnerable populations were not involved.

## Author Contributions

C-YL, CT, and DG performed the experiments, analyzed the data, and wrote the manuscript. R-LW, H-ZZ, and MN collected and prepared samples. R-YL and C-EZ performed the analyses. R-SL amended the paper. X-HX, M-HY, and J-BW designed the study and amended the paper.

## Conflict of Interest Statement

The authors declare that the research was conducted in the absence of any commercial or financial relationships that could be construed as a potential conflict of interest.

## Footnotes

1 www.genome.jp/kegg/

2 http://www.metaboanalyst.ca/

3 https://metlin.scripps.edu/
