# Supplementary material for: Metabolomic Study on Idiosyncratic Liver Injury Induced by Different Extracts of Polygonum multiflorum in Rats Integrated with Pattern Recognition and Enriched Pathways Analysis
Source: Front Pharmacol. 2016 Dec 15;7:483. doi: 10.3389/fphar.2016.00483 (PMC5156827; doi:10.3389/fphar.2016.00483)
Supplement: Supplementary file 1 [file Presentation_1.pdf]

Supplementary materials for:

Metabolomic study on idiosyncratic liver injury induced by different extracts of *Polygonum multiflorum* in rats integrated with pattern recognition and enriched pathways analysis

Chun-yu Li[1,2‡], Can Tu[2,4‡], Dan Gao[1,2‡], Rui-lin Wang[3], Hai-zhu Zhang[2,4],Ming Niu[2], Rui-yu Li[2], Cong-en Zhang[2], Rui-sheng Li[5], Xiao-he Xiao, Mei-hua Yang[1*], Jia-bo Wang[2*]

[1] Institute of Medicinal Plant Development, Chinese Academy of Medical Sciences &Peking Union Medical College, China, [2] China Military Institute of Chinese Medicine, 302 Military Hospital, China, [3] Integrative Medical Center, 302 Military Hospital, China, [4] School of Pharmacy, Chengdu University of Traditional Chinese Medicine, China, [5] Research Center for Clinical and Translational Medicine, 302 Hospital of People's Liberation Army, China.

1.Chemical compositions determination of the different extracts of *Polygonum multiflorum*

The main consituents in the $CHCl_3$、EtOAc and Residue extracts of *Polygonum multiflorum* used in the experiment were analyzed by UHPLC. The sample was filtered through a syringe filter(0.22μm) and transferred into the sampling vial pending UHPLC analysis that were performed on a Agilent Acquity ultraperformance liquid chromatography system with column oven temperature maintained at 30℃, using an ZORBA×300 SB-C18column (2.1 mm×100 mm i.d., 1.7μm particle size) (Agilent, USA). UV detection was performed at 280nm. The mobile phase was constituted by solvent A (0.1% formic acid in water) and solvent B (acetonitrile). The flow rate was 0.2 mL min^{-1} with a linear gradient at the following conditions: 0～5 min, 5% B; 5～6 min,32%～55% B; 6～12 min,55～85% B; 12～13 min, 85%～90%B; 13～15 min, 90% B. The injection volume was 3μL.

The 2,3,5,4'-tetrahydroxystilbene-2-O-β-D-glucopyranoside(TSG), emodin-8-O-β-D-glucopyranoside and emodin reference substances were provided by the Chengdu Chroma-Biotechnology Co., Ltd and the purity of all these compounds were higher than 98.0%. **FIGURE S1 A** shows the chromatogram of 2,3,5,4'-tetrahydroxystilbene-2-O-β-D-glucopyranoside, emodin-8-O-β-D-glucopyranoside and emodin standard mixture. **FIGURE S1 B, C and D** show the UHPLC profile of EtOAc, $CHCl_3$ and Residue extract, respectively. The results of chromatography analysis showed that the major chemical compositions of EtOAc extract were stilbenes(TSG), the other was slight emodin-8-O-β-D-glucopyranoside. In addition, anthranoid derivatives, such as emodin, were mainly contained in $CHCl_3$ extract. And the RE extract contained neither anthraquinones nor stilbenes.

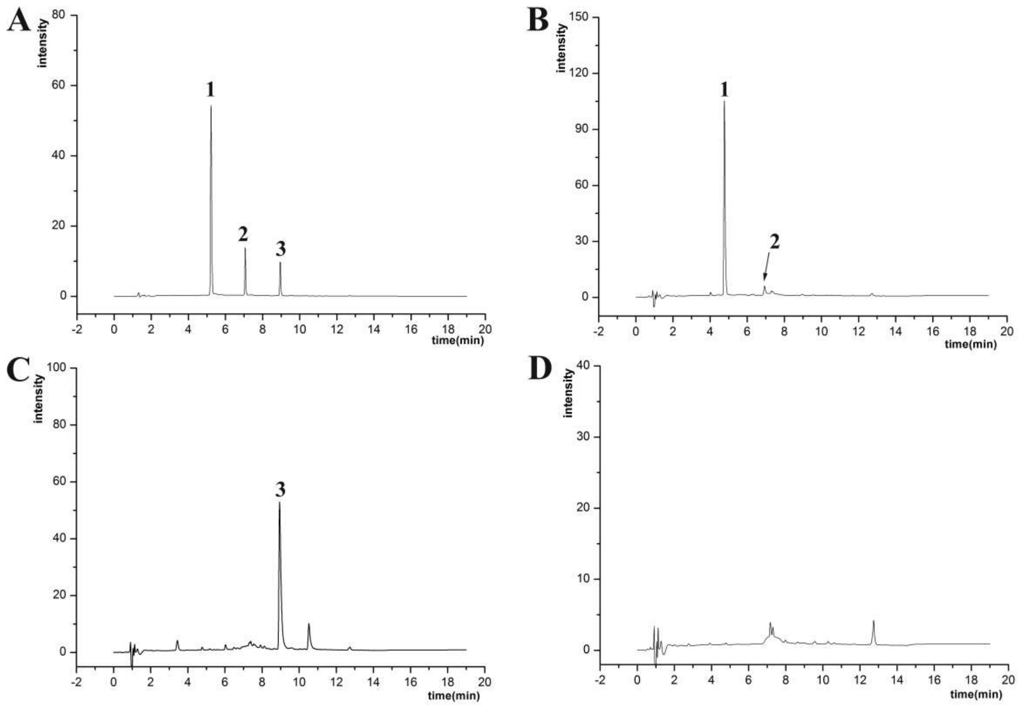

FIGURE S 1 Ultra-high performance liquid chromatography of different extracts of *Polygonum multiflorum.* (**A**) Chromatogram of 2,3,5,4’-tetrahydroxystilbene-2-O-β-D-glucopyranoside, emodin-8-O-β-D-glucopyranoside and emodin standard mixture. (**B**)UHPLC profile of EtOAc extract. **(C)** UHPLC profile of $CHCl_3$ extract. **(D)** UHPLC profile of Residue extract. 1. 2,3,5,4’-tetrahydroxystilbene-2-O-β-D- glucopyranoside;2. emodin-8-O-β-D-glucopyranoside; 3. emodin

2. Parameters of PCA, heatmap and OPLS-DA models and the results of OPLS-DA analysis based on data derived from ESI+ mode

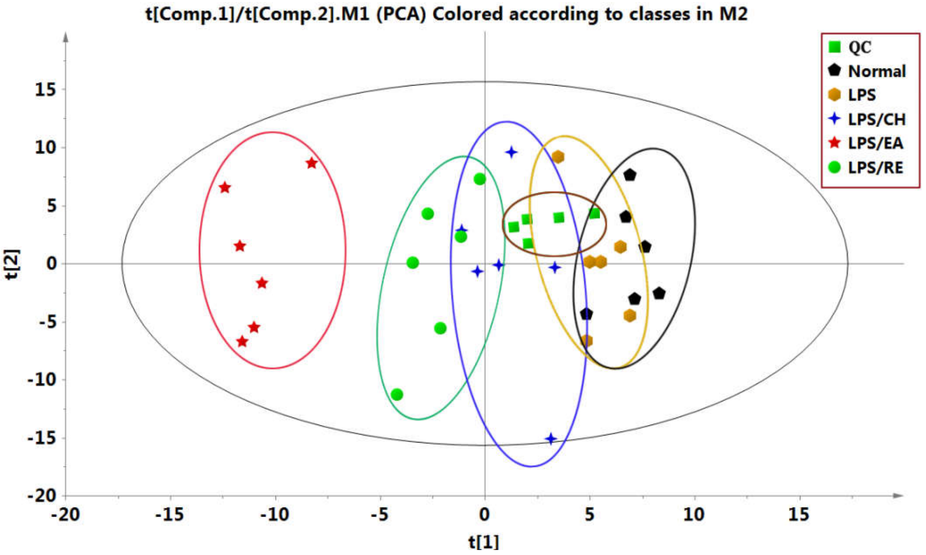

FIGURE S2 PCA score plots of different extracts of *Polygonum multiflorum* by UPLC-HDMS in positive ESI mode.

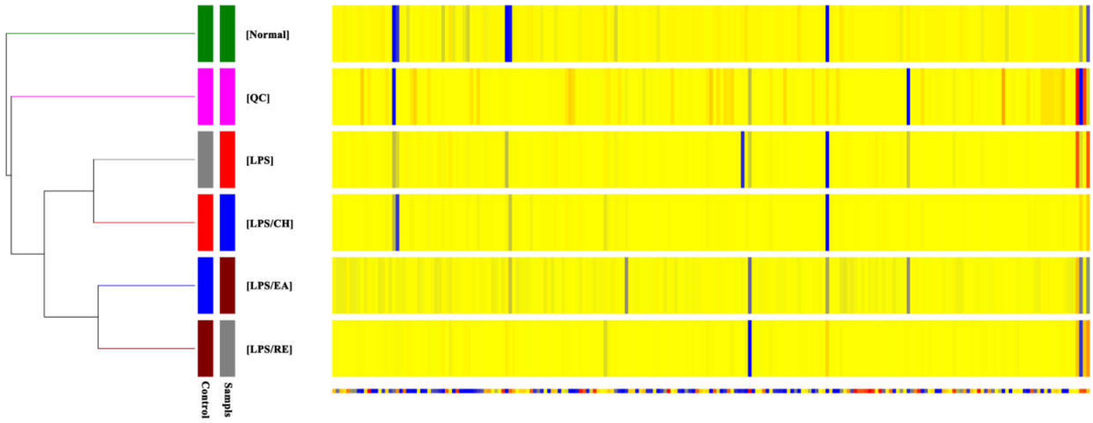

FIGURE S3 Heatmap visualization for different extracts of Polygonum multiflorum. Rows: samples; Columns: metabolites(ESI+ mode)

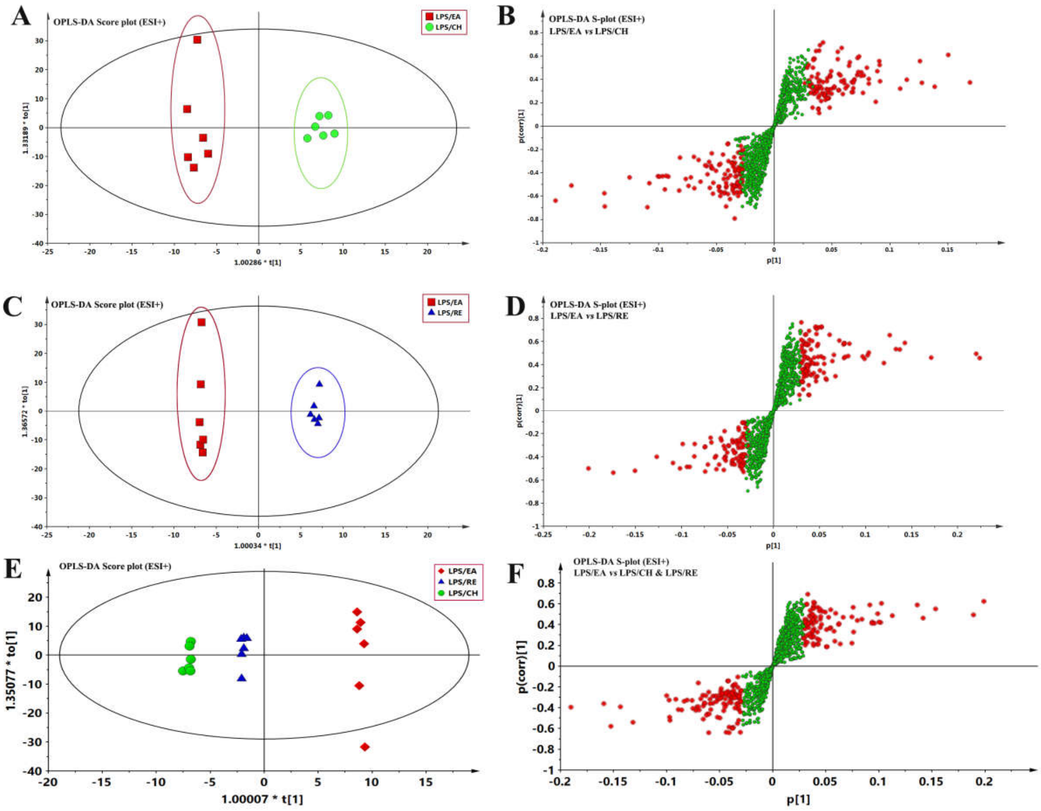

FIGURE S4 OPLS-DA analysis of the data generated from the ESI+ mode. S-score plots constructed from the supervised OPLS analysis of serum(B,D and F), the axes that are plotted in the S-plot from the predictive component are p1 *vs* p(corr)1, representing the magnitude and reliability respectively. Metabolite ions with VIP value >1 were marked with a red square. (A, B) displays the result of OPLS-DA model (M2) using the data from the LPS/EA and LPS/CH groups in ~~ESI-~~ Note:It should be "ESI+" mode, (C, D) displays the result of OPLS-DA model (M3) using the data from the LPS/EA and LPS/RE groups in ~~ESI-~~ Note:It should be "ESI+" mode, (E, F) displays the result of OPLS-DA model (M4) using the data from the LPS/EA , LPS/CH and LPS/RE groups in ESI+ mode.

3. Pathway analysis of potential marker metabolites for different extracts of *Polygonum multiflorum* treatment

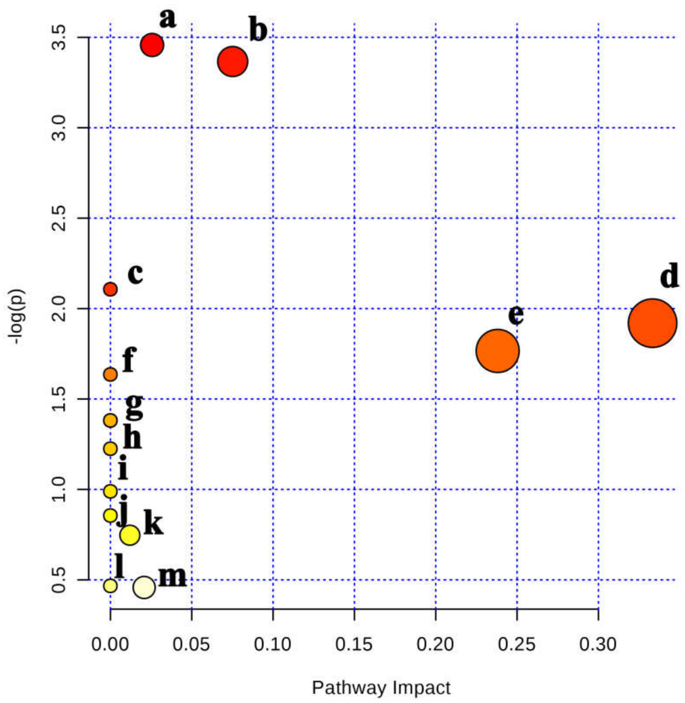

Figure S5 Summary of pathway analysis with MetaboAnalyst 3.0. a, Citrate cycle (TCA cycle); b, Sphingolipid metabolism; c, Vitamin B6 metabolism; d. Valine, leucine and isoleucine biosynthesis; e, Nicotinate and nicotinamide metabolism; f. Pantothenate and CoA biosynthesis; g. Propanoate metabolism; h. Butanoate metabolism; i. Alanine, aspartate and glutamate metabolism;j. Glycine, serine and threonine metabolism; k. Valine, leucine and isoleucine degradation; l. Arginine and proline metabolism; m. Purine metabolism

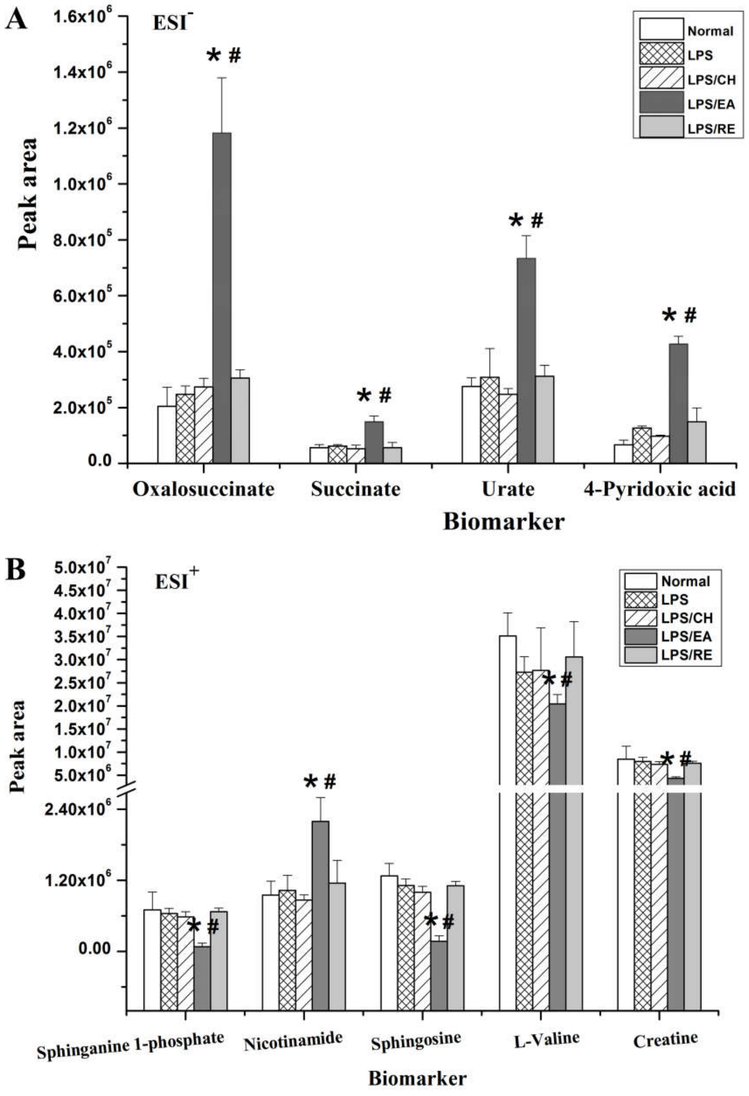

Figure S6 Graphical representation of potential markers among the Normal, LPS, LPS/EA , LPS/CH and LPS/RE groups. (A) In negative ion mode, (B) in positive ion mode. Bars in the Fig. represent the mean relative metabolite concentration and standard deviations. Compared with the LPS/CH group, * represents $p < 0.05$; Compared with the LPS/RE group,# represents $p < 0.05$.

Table S1. Parameters of PCA and OPLS-DA models

Model	No.[b]	R^2X_{cum}[a]	R^2Y_{cum}[a]	Q^2Y_{cum}[a]
PCA (ESI^-)	7	0.705	—	0.527
PCA (ESI^+)	8	0.803	—	0.579
OPLS-DA (ESI^-) LPS/EA *vs* LPS/CH	3	0.586	0.990	0.661
OPLS-DA (ESI^-) LPS/EA *vs* LPS/RE	2	0.631	0.996	0.503
OPLS-DA (ESI^-) LPS/EA *vs* LPS/CH& LPS/RE	4	0.606	0.812	0.502
OPLS-DA (ESI^+) LPS/EA *vs* LPS/CH	3	0.689	0.991	0.79
OPLS-DA (ESI^+) LPS/EA vs LPS/RE	3	0.623	0.927	0.553
OPLS-DA (ESI+) LPS/EA vs LPS/CH& LPS/RE	6	0.772	0.867	0.537

a R^2X_{cum} and R^2Y_{cum} are the cumulative modeled variation in X and Y matrix, respectively. Q^2Y_{cum} is
the cumulative predicted variation in Y matrix.
b Number of components.

Table S2 Result from pathway analysis with MetaboAnalyst 3.0

Table S2 Result from pathway analysis with MetaboAnalyst 3.0

Pathway name	Total	Hits	Raw *p*	-log(*p*)	FDR	Impact
Citrate cycle (TCA cycle)	20	2	0.0315	3.4576	1.00	0.03
Sphingolipid metabolism	21	2	0.0345	3.3660	1.00	0.08
Vitamin B6 metabolism	9	1	0.1216	2.1068	1.00	0.00
Valine, leucine and isoleucine biosynthesis	11	1	0.1467	1.9195	1.00	0.33
Nicotinate and nicotinamide metabolism	13	1	0.1711	1.7658	1.00	0.24
Pantothenate and CoA biosynthesis	15	1	0.1948	1.6360	1.00	0.00
Propanoate metabolism	20	1	0.2512	1.3813	1.00	0.00
Butanoate metabolism	20	1	0.2512	1.3813	1.00	0.00
Alanine, aspartate and glutamate metabolism	24	1	0.2937	1.2252	1.00	0.00
Glycine, serine and threonine metabolism	32	1	0.3719	0.9893	1.00	0.00
Valine, leucine and isoleucine degradation	38	1	0.4250	0.8557	1.00	0.00
Arginine and proline metabolism	44	1	0.4739	0.7469	1.00	0.01
Purine metabolism	68	1	0.6326	0.4579	1.00	0.02

Total is the total number of compounds in the pathway; the Hits is the actually matched number from the user uploaded data; the Raw p is the original p value calculated from the enrichment analysis; the FDR p is the p value adjusted using False Discovery Rate; the Impact is the pathway impact value calculated from pathway topology analysis.
